# Identification and in vivo characterization of a brain-penetrating nanobody

**DOI:** 10.1186/s12987-020-00226-z

**Published:** 2020-10-14

**Authors:** Y Wouters, T Jaspers, B De Strooper, M Dewilde

**Affiliations:** 1 VIB Center for Brain & Disease Research, Campus Gasthuisberg O&N4, Herestraat 49, box 602, B-3000 Leuven, Belgium; 2 Laboratory for the Research of Neurodegenerative Diseases, Department of Neurosciences, Leuven Brain Institute (LBI), KU Leuven, B-3000 Leuven, Belgium; 3 UK Dementia Research Institute, University College London, London, UK; 4 VIB Discovery Sciences, B-3000 Leuven, Belgium; 5 Present Address: Laboratory for Therapeutic and Diagnostic Antibodies, Department of Pharmaceutical and Pharmacological Sciences, KU Leuven, B-3000 Leuven, Belgium

**Keywords:** Nanobody, VHH, Transferrin receptor, Neurotensin, Blood–brain barrier, Receptor-mediated transcytosis

## Abstract

**Background:**

Preclinical models to determine blood to brain transport ability of therapeutics are often ambiguous. In this study a method is developed that relies on CNS target-engagement and is able to rank brain-penetrating capacities. This method led to the discovery of an anti-transferrin receptor nanobody that is able to deliver a biologically active peptide to the brain via receptor-mediated transcytosis.

**Methods:**

Various nanobodies against the mouse transferrin receptor were fused to neurotensin and injected peripherally in mice. Neurotensin is a neuropeptide that causes hypothermia when present in the brain but is unable to reach the brain from the periphery. Continuous body temperature measurements were used as a readout for brain penetration of nanobody-neurotensin fusions after its peripheral administration. Full temperature curves were analyzed using two-way ANOVA with Dunnett multiple comparisons tests.

**Results:**

One anti-transferrin receptor nanobody coupled to neurotensin elicited a drop in body temperature following intravenous injection. Epitope binning indicated that this nanobody bound a distinct transferrin receptor epitope compared to the non-crossing nanobodies. This brain-penetrating nanobody was used to characterize the in vivo hypothermia model. The hypothermic effect caused by neurotensin is dose-dependent and could be used to directly compare peripheral administration routes and various nanobodies in terms of brain exposure.

**Conclusion:**

This method led to the discovery of an anti-transferrin receptor nanobody that can reach the brain via receptor-mediated transcytosis after peripheral administration. This method could be used to assess novel proteins for brain-penetrating capabilities using a target-engaging readout.

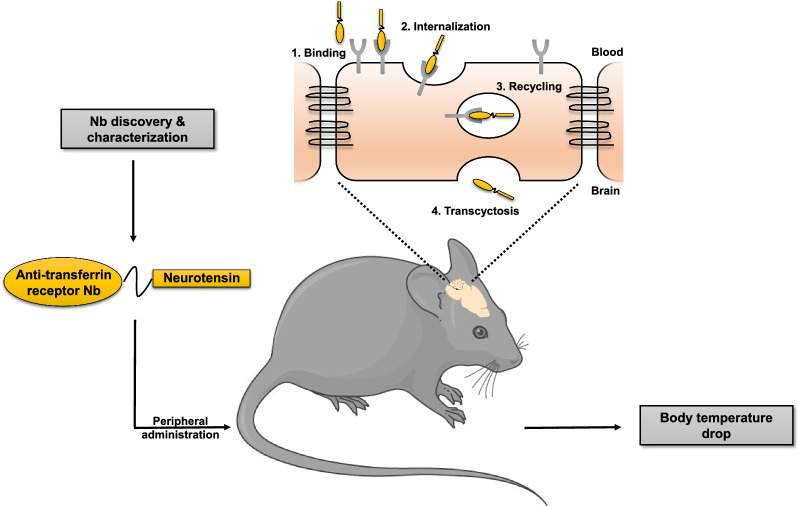

## Background

Delivery of biologics to the central nervous system (CNS) has been a major challenge. This is partly due to the fact that the CNS is physically separated from the periphery by several barriers, including the blood–brain barrier (BBB), a monolayer of endothelial cells supported by astrocytes and pericytes [1]. It is estimated that only 0.1% of circulating macromolecules is able to reach the brain parenchyma, which severely limits the use of biologics to treat CNS-related diseases [2].

Transcytosis pathways involved in the delivery of essential nutrients have been explored for delivery of drugs to the brain. Nutrients are able to cross the BBB via specific receptors expressed on the luminal side of brain endothelial cells via receptor-mediated transcytosis (RMT). So-called Trojan Horse approaches exploit this mechanism. Therapeutic biologics are coupled to receptor-targeting entities such as peptides or antibodies binding to these nutrient receptors to shuttle them to the brain [3, 4]. Regardless intense research of RMT [5] during the past 30 years, only two drug candidates successfully completed phase 1–2 clinical trials [6, 7]. Moreover, currently there are no therapeutic proteins approved for clinical use that cross the BBB to exert their effect [8].

Nanobodies (also called single-domain antibodies or VHHs) could be an interesting addition to the existing Trojan Horse delivery methods. Nanobodies are derived from the variable domain of the heavy-chain-only antibodies found in camelids [9, 10]. They recognize antigens with similar affinities and specificity as monoclonal antibodies and can be easily fused to a wide variety of compounds [11–15]. Nanobodies have been used in various fields, ranging from therapeutic to biochemistry applications [10, 16–18].

Many reports have claimed access of nanobodies to the brain. Due to their small size and often cationic charge, nanobodies are able to fuse with the negatively charged cell membrane which can lead to brain uptake via adsorptive-mediated transcytosis [19]. This mechanism entirely relies on the charge of the brain-penetrating entity. Fusion to a therapeutic compound changes this charge which then alters crossing efficiency [20–22]. Since small changes or fusions of various entities to nanobodies that reach the brain via adsorptive-mediated transcytosis can alter their crossing capabilities, such nanobodies are not suitable as a Trojan Horse delivery systems. Instead, nanobodies that utilize RMT to deliver drugs to the CNS could be more successful Trojan Horses.

One of the most investigated RMT targets is the transferrin receptor (TfR), which is highly expressed on brain endothelial cells [5, 23]. Anti-TfR monoclonal antibodies deliver therapeutics to the brain in rats [24, 25], mice [26, 27], monkeys [4] and humans [7]. Also Tfr antibody-fragments, such as single-chain variable fragments [28–30], Fab fragments [31] or dual variable domain immunoglobulins [32], are able to facilitate blood to brain transport. Very recently, two studies have showed that engineering the Fc fragment of a monoclonal antibody to target the TfR resulted in more brain uptake of various therapeutic proteins [33, 34]. This previous research opens the door for the discovery of brain-penetrating, anti-TfR nanobodies. In order to select potential Trojan Horse nanobodies (meaning nanobodies reaching the brain via RMT and not by adsorptive-mediated transcytosis), unequivocal preclinical evidence for transfer to the brain has to be delivered.

A reliable method to demonstrate CNS target-engagement is inhibition of beta-secretase 1 (BACE1). BACE1 is an enzyme that cleaves the amyloid precursor protein (APP) in neuronal endosomes leading to the generation of amyloid-β peptides (Aβ) [35, 36]. Here, brain-penetrating moieties fused to a BACE1 inhibiting entity are intravenously injected into animal species, after which the brains are harvested and homogenized. A decrease in central Aβ levels, measured by ELISA, indicates BBB crossing [33, 37–40]. While this proves good evidence for functional brain targeting this method requires for each measurement point the use of multiple mice [41]. Moreover the further processing of brain extracts and ELISAs are time consuming and expensive, prohibiting large screening efforts.

In order to improve the robustness of preclinical in vivo CNS research and reduce the number of animals needed for proof-of-concept, a method is needed that demonstrates brain uptake by target engagement, is unambiguous with regard to brain target, and, finally, allows for reuse of laboratory animals. Here, we explore whether it is possible to use a nanobody to reach the brain using new nanobodies raised against the mouse Transferrin receptor TfR (mTfR), a known receptor-mediated transcytosis target. As a readout, we coupled the nanobodies to NT, a neuropeptide that elicits hypothermia after binding to the NT receptor (NTSR1) expressed in the CNS [42]. Since this receptor is a G-protein-coupled receptor (GPCR) located in the cell membrane of hypothalamic neurons [43], it can interact with molecules in the interstitial fluid. In contrast to intracerebroventricular administered NT, intravenously administered NT is not able to elicit a hypothermic response [44]. Therefore, the observed hypothermic effect after intravenous injection of nanobody-NT fusions is direct evidence of BBB transport facilitated by that particular nanobody. Moreover, this method assesses the brain penetrating capabilities of the generated nanobodies after a single IV injection and without the need to sacrifice the animal, making it possible to use the animal multiple times. The developed method led to the discovery of the first nanobodies that can deliver a cargo (NT) to the brain via the TfR.

## Materials and methods

### Nanobody library generation

Nanobodies targeting the mouse transferrin receptor were obtained in collaboration with the VIB Nanobody Core. A llama was immunized with the extracellular domain of the mouse transferrin receptor (50741-M07H-100, Sino Biological) by subcutaneous injections on days 0, 7, 14, 21, 28 and 35. The first injection contained 100 µg protein followed by five injections with 70 µg protein each. On day 40 a blood sample of 100 ml was collected and peripheral blood lymphocytes were isolated. The nanobody library was cloned into a phagemid vector as previously prescribed [45]. Briefly, total RNA from peripheral blood lymphocytes was used as template for first strand cDNA synthesis with oligodT primer. This cDNA was used to amplify the nanobody-encoding open reading frames by PCR, digested with PstI and NotI, and cloned into the phagemid vector pMECS. The library was transformed into electro-competent *E.coli* TG1 cells, which resulted in 10^8^ independent transformants, of which 85% contained the vector with a right insert size.

### Isolation of anti-mTfR nanobodies

To select anti-mTfR nanobodies, two rounds of in solution selections were performed with 100 and 50 nM biotinylated mTfR (50741-M07H-100, Sino Biological), respectively. After the second round the library was subcloned into an expression vector (pBDS100, a modified pHEN6 vector with an OmpA signal peptide and a C-terminal 3xFlag/6xHis tag) [46]. The expression library was used to transform TG1 *E.coli* after which nanobodies were expressed from single colonies. These nanobodies were screened for direct binding to the biotinylated mTfR using the AlphaScreen Histidine Detection Kit (6760619 M, Perkin Elmer). The hits were sequenced and clustered according to sequence homology. One representative of each sequence cluster was recloned into the NT vector (pBDS100 with C-terminal NT), expressed and purified following the protocol by Pardon et al. [45]. In total, 7 nanobodies were successfully recloned and expressed.

### Generation of affinity variants

Single amino acid substitutions in the CDR3 region of Nb62 were generated following the protocol of Kille et al. [47]. In short, 13 separate PCRs were performed using the Phusion High-Fidelity PCR Kit (F553S, Thermo Scientific) and purified via agarose gel electrophoresis and a QIAquick Gel Extraction Kit (28704, Qiagen). The methylated template DNA constructs were removed by *DpnI* digestion and the products were purified using QIAquick PCR Purification Kit (28104, Qiagen). Next, the plasmids were mixed in equimolar amounts and transformed into TG1 *E.coli*. Screening of single colonies was performed as described above.

### In vitro binding

#### Bio-layer interferometry

Binding of the purified nanobodies to various forms of the transferrin receptor was assessed using an Octet RED96 (Forté Bio/Molecular Devices). Initially, streptavidin (SA) biosensor tips (18–5020, Forté Bio/Molecular Devices) were pre-wet for minimally 10 min in 1xPBS, after which they were dipped in biotinylated TfR (1 µg/ml in 1xPBS). Next, the tips were sequentially submerged in baseline wells (1xPBS), dissociation wells (1xPBS), nanobodies (100 nM in 1xPBS) and dissociation wells. Sensorgrams were generated using the Forté Bio Octet RED analysis software (Forté Bio/Molecular Devices).

#### ELISA

Nunc maxisorp™ 96-well plates (44-2404-21, Thermo Fisher) were coated with 0.1 µg of mTfR in 100 µl PBS per well and incubated overnight at 4 °C. The next day, plates were washed 5 times with 200 µl PBS (0,05% tween-20) and blocked with 150 µl PBS (0,1% casein) for 1 h at room temperature. Then, 50 µl of dilution series from 10 μM to 0,1 nM in blocking buffer per nanobody were added and incubated for 1 h at room temperature, followed by 5 washes. Subsequently, 50 µl of mouse anti-FLAG M2 monoclonal antibody (F3165, Sigma, 1 in 20.000 diluted in PBS/casein) was added for 1 h at room temperature, followed by 5 washes and 50 µl of horseradish peroxidase-conjugated goat anti-mouse antibody (P0447, DAKO, 1 in 20.000 diluted in PBS/casein) for 1 h at room temperature. Next, 5 washes were performed and the reaction was developed using 100 µl of developing solution (10 mL sodium acetate (pH 4.9), 100 µl 3,3′,5,5′-Tetramethylbenzidine, 10 µl hydrogen peroxide) and stopped upon blue color formation using 100 µl sulfuric acid (2 N). Absorbance at 450 nm was measured using an EnVision® multimode plate reader (Perkin Elmer).

### In vivo binding

#### Animals

All animal experiments were conducted according to protocols approved by the local Ethical Committee of Laboratory Animals of the KU Leuven (governmental license LA1210579, ECD project number 040/2016) following governmental and EU guidelines. The in vivo experiments were performed using both male and female mice ages 2–6 months. The sample size (n = 3) was calculated by a continuous endpoint, two independent sample groups using the following parameters: mean ∆T group 1 = 0 °C, standard deviation on ∆T ± 0.5 °C, mean ∆T group 2 = 2.5 °C, *p* value ≤ 0.05 and power = 0,8.

#### Anipill® implantation

To automatically measure body temperature of socially housed mice, the Anipill® (BodyCap) system was implanted in TLR4^−/−^ mice. These mice are resistant to endotoxins, which would prevent potential BBB opening by residual endotoxins. TLR4^−/−^ mice were injected with buprenorphine (0,05 mg/kg, SC) an hour before Anipill® implantation, followed by lidocaine (6 mg/kg, SC under the scalp) as local analgesia five minutes before implantation. The mice were induced with 5% isoflurane and were placed in the stereotactic frame with 1–2% isoflurane. The abdomen was opened and the Anipill® was implanted. Next, the muscle layer was sutured with resorbable sutures and the skin was closed with surgical staples. Then, 500 µl of saline was injected subcutaneously and the animals were allowed to recovery under a heating lamp, followed by an additional injection of buprenorphine (0.1 mg/kg) 6 h later. The Anipill® implantation was performed at least 1 week prior to any experiment.

#### ICV injections

The intracerebroventricular injections were performed as previously described [48] using the following stereotactic coordinates: AP—0.1 mm, ML—1.0 mm, and DV—3.0 mm (from the skull). After minimum 1 week of recovery, 2 µl of saline or sample was injected slowly via a Hamilton syringe into the lateral ventricle. Body temperature was monitored every 15 min using the Anipill® system.

#### IV/IP/SC injections

For intravenous injections, the mouse was put in a restrainer and the tail was heated in warm water between 42 and 48 °C. Then, nanobodies were injected in the tail vein at volumes between 100 and 180 µl. For intraperitoneal and subcutaneous injections, the mouse was immobilized and 100 µl of the designated nanobody was injected [49]. Body temperature was monitored every 15 min using the Anipill® system.

## Results

### Generation and expression of anti-mouse transferrin receptor nanobodies

In order to find BBB crossing nanobodies, an alpaca was immunized with the extracellular domain of the mouse transferrin receptor. Peripheral blood lymphocytes were isolated, total RNA was extracted and used as template for first strand cDNA synthesis. Next, the nanobody-encoding open reading frames were amplified by a nested polymerase chain reaction, cloned into a phagemid vector and transformed into TG1 *E.coli*. This yielded a nanobody library with a functional size of about 10^8^ independent transformants. To enrich for anti-mTfR-specific nanobodies, two consecutive rounds of phage selection were completed until a 100-fold increase in binding phages was observed compared to the negative control (no antigen) phage selection by phage titration. Then, the libraries were subcloned into an expression vector, individual clones were picked, expressed and crude extracts were screened using AlphaScreen technology for specific binding to the extracellular domain of the mTfR. In total 282 clones were screened of which 82 nanobodies were able to bind the mTfR. Based on sequence similarity they were classified into 11 different sequence clusters. A representative nanobody was selected for each cluster and 7 nanobodies were successfully re-cloned and expressed as soluble nanobodies with a C-terminal 3XFLAG and a hexahistidine tag followed by neurotensin [50]. Expression was directed to the periplasm after which extraction through osmotic shock was performed. The nanobodies were purified from the resulting extract using immobilized metal-ion affinity chromatography.

### In vitro characterization of the anti-mTfR nanobodies

**Fig. 1:**
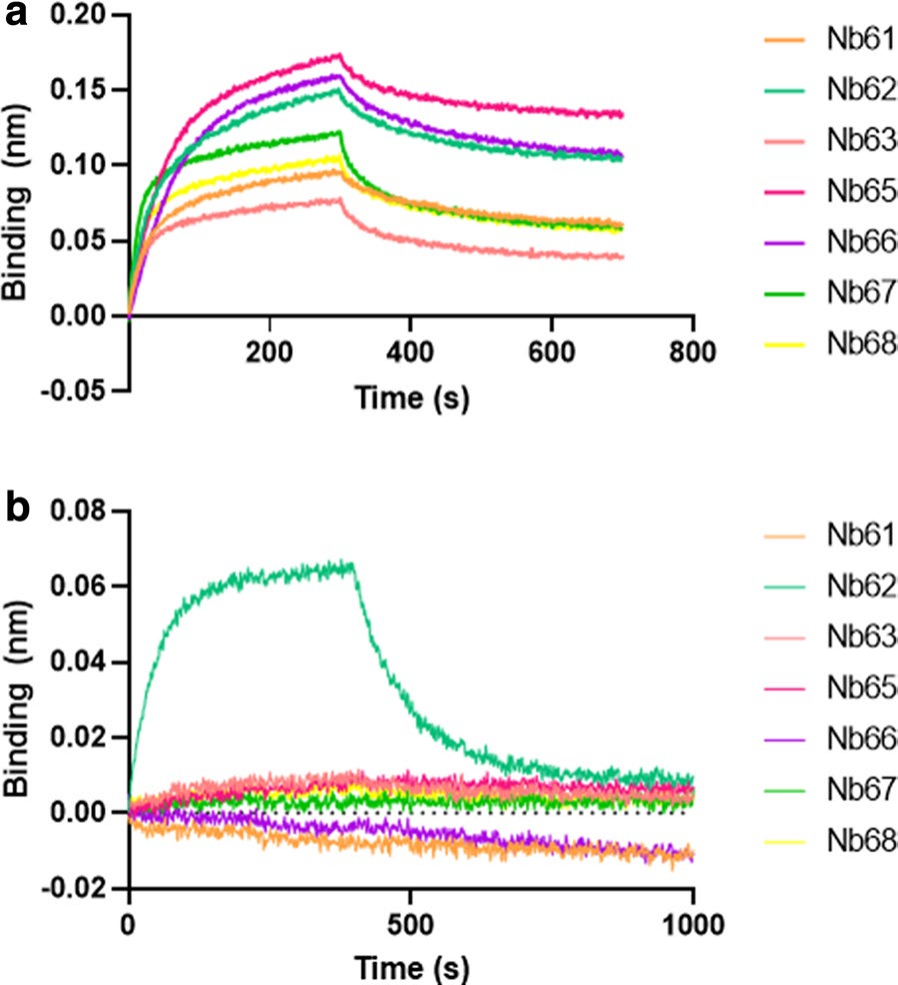
Binding of anti-mTfR nanobodies to mouse and chimeric human TfR. Binding of anti-mTfR to biotinylated mTfR **a** or biotinylated chimeric human TfR with mouse apical domain **b** immobilized on streptavidin biosensors. Biosensors were dipped in 1 µg/ml TfR, followed by 100 nM of nanobody. All nanobodies bind mTfR (A) of which only one, Nb62, was also able to bind the chimeric TfR (B)

The protein identity and integrity was confirmed using mass spectrometry (data not shown). Next, binding to the mTfR was confirmed by bio-layer interferometry using the Octet system (Fig. 1a). At the same time binding to the human TfR was also assessed, but none of the nanobodies turned out to be cross-reactive. This lack of cross-reactivity has already been observed for anti-TfR antibodies [51]. We took advantage of this lack of cross reactivity to perform epitope binning. Since currently known brain penetrating monoclonal antibodies bind the apical domain of the TfR [31], a chimeric receptor was generated where the human apical domain was exchanged for the mouse sequence (custom designed, expressed and purified by GenScript Biotech). Again, the Octet system was used to assess binding of the anti-mTfR nanobodies (Fig. 1b). As can be seen, only Nb62 was able to bind the chimeric receptor with mouse apical domain.

### In vivo validation of anti-mTfR nanobodies

**Fig. 2:**
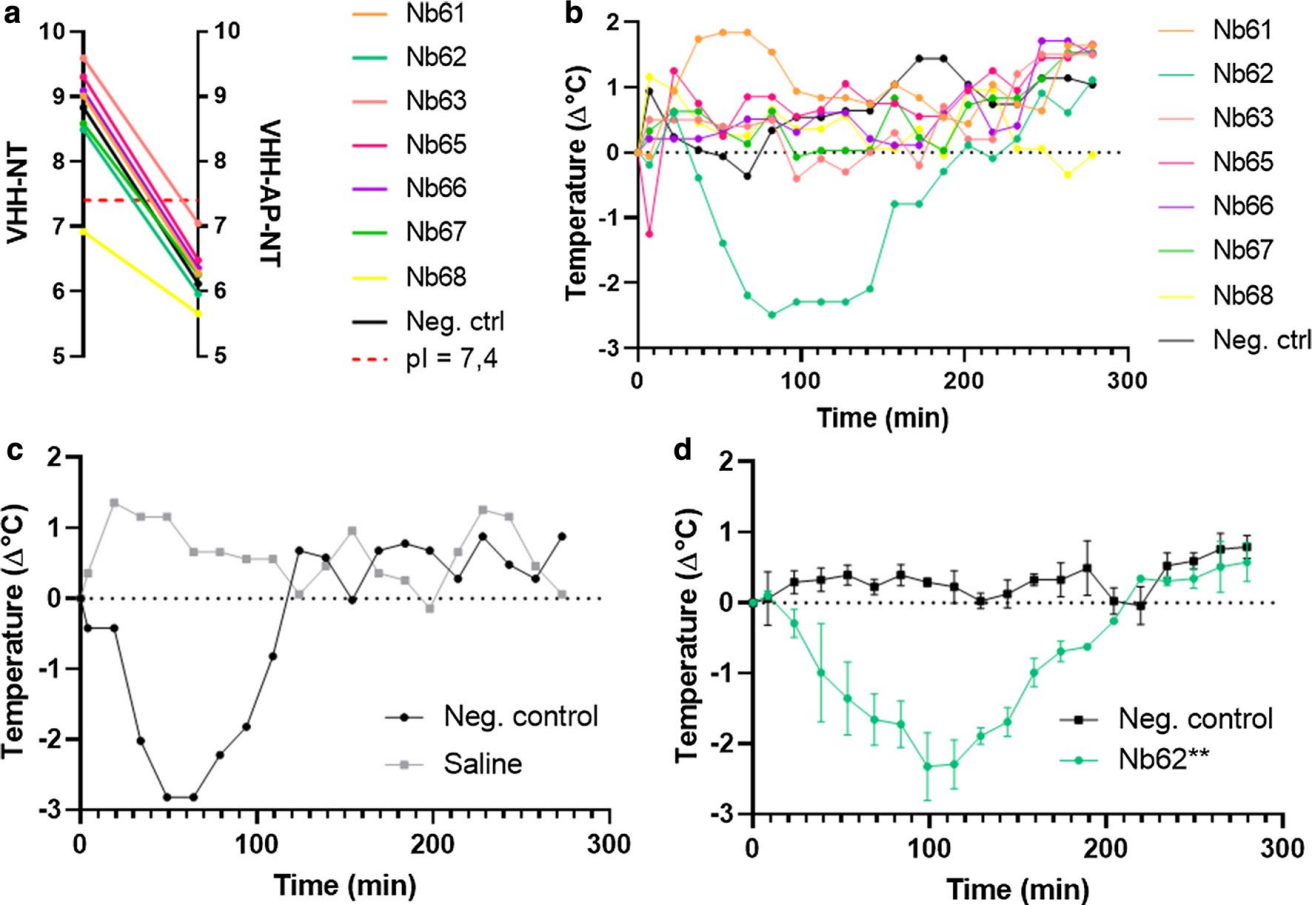
Evaluation of anti-mTfR nanobodies for receptor-mediated transcytosis. **a** Theoretical pIs of the anti-mTfR nanobodies fused to NT, with or without an incorporated acidic peptide, calculated based on their primary sequence by CLC Main Workbench 8.1. A pI corresponding to no net charge under physiological conditions is indicated by the red dotted line. **b** TLR4^−/−^ mice body temperature measurements after 250 nmol/kg intravenous injections of the indicated nanobody fused to NT. **c** TLR4^−/−^ mice body temperature measurements after 5 pmol ICV injection of the negative control nanobody fused to NT. **d** TLR4^−/−^ mice body temperature measurements after 250 nmol/kg IV injections of Nb62 or negative control (**p = 0.004). Bar graphs represent mean ± SEM (n = 3 per group, *p* = 0.004). Statistical test: two-way ANOVA with Dunnett multiple comparisons test compared to negative control nanobody

In vitro binding however does not mean in vivo crossing. In order to find functionally active anti-mTfR nanobodies, an in vivo screening platform was designed. This platform relies on the NT linked to the nanobodies. NT is a neuropeptide that does not reach the brain parenchyma on its own [50] but lowers the body temperature when it is present in the CNS. If the nanobody-NT fusion would cause a drop in temperature upon IV injection, this would imply that the NT has been transported to the brain by the nanobody. Since nanobodies with a basic isoelectric point (pI) can cross the BBB via adsorptive-mediated transcytosis [19, 22], we calculated the theoretic pI of all nanobody-NT fusions and took care to incorporate an acidic peptide (AP) between the nanobody and NT sequence so that all overall pIs were acidic, ranging from 5.66 to 7.05 (Fig. 2a). These acidic anti-mTfR nanobodies and a negative control nanobody (raised against green fluorescent protein) were intravenously injected in order to assess their brain-penetrating potential. From the seven injected nanobodies, one (Nb62) was able to elicit a drop in body temperature, which indicated target engagement of NT in the CNS (Fig. 2b). In order to ensure that the lack of decrease in body temperature of the negative control was due to the fact that it did not enter the brain versus a loss of NT function, it was injected directly into the ventricle (Fig. 2c). Here the negative control showed a drop in body temperature, indicating (together with the mass spectrometry data) that the construct contained a functional NT sequence. Next, the brain-penetrating effect of Nb62 was confirmed in three independent mice and showed a significant decrease in body temperature compared to the negative control (Fig. 2d).

### Hypothermic effect by nanobody-NT fusions is dose-dependent

**Fig. 3:**
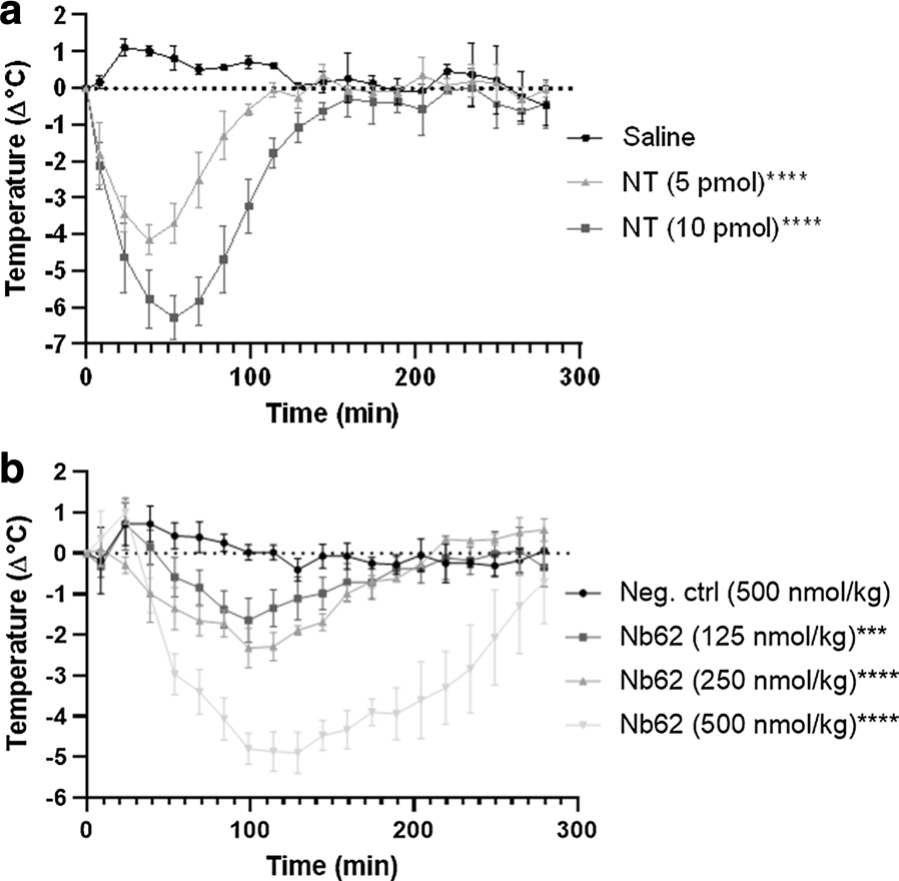
Evaluation of the dose–response relationship of NT. **a** TLR4^−/−^ mice body temperature measurements after ICV injections of the indicated doses of NT or saline (*p = 0.0249, ****p < 0.0001). Bar graphs represent mean ± SEM (n = 3 per group for saline and NT (5 pmol) and n = 2 for NT (10 pmol)). **b** TLR4^−/−^ mice body temperature measurements after intravenous injections of the indicated doses of Nb62 or negative control nanobody fused to NT (****p < 0.0001). Bar graphs represent mean ± SEM (n = 3 per group). Statistical test: two-way ANOVA with Dunnett multiple comparisons test compared to saline or negative control nanobody

We next determined whether the method allows dose-dependent correlations as this could be used to directly compare the brain-penetrating efficiency of nanobodies and their modifications. Since NT does not reach the brain by itself, it was injected in the lateral ventricle (ICV) at different doses. As can be seen from Fig. 3a, the drop in body temperature doubles when the dose increases from 5 to 10 pmol. This means that the extent of the hypothermic effect is positively correlated with the amount of NT present in the CNS. To determine if this dose–response relationship translates to brain penetration, different doses of Nb62 were injected via the tail vein (Fig. 3b). Again, the decrease in body temperature positively correlates with the amount of injected Nb62. Moreover, the negative control at 500 nmol/kg did not have a hypothermic effect, indicating that it was not able to reach the CNS. This shows that the hypothermic effect caused by NT can be correlated to the relative amount of nanobody that has actively reached the brain.

### NT in vivo screening method can distinguish brain-penetrating efficiency of nanobodies

**Fig. 4:**
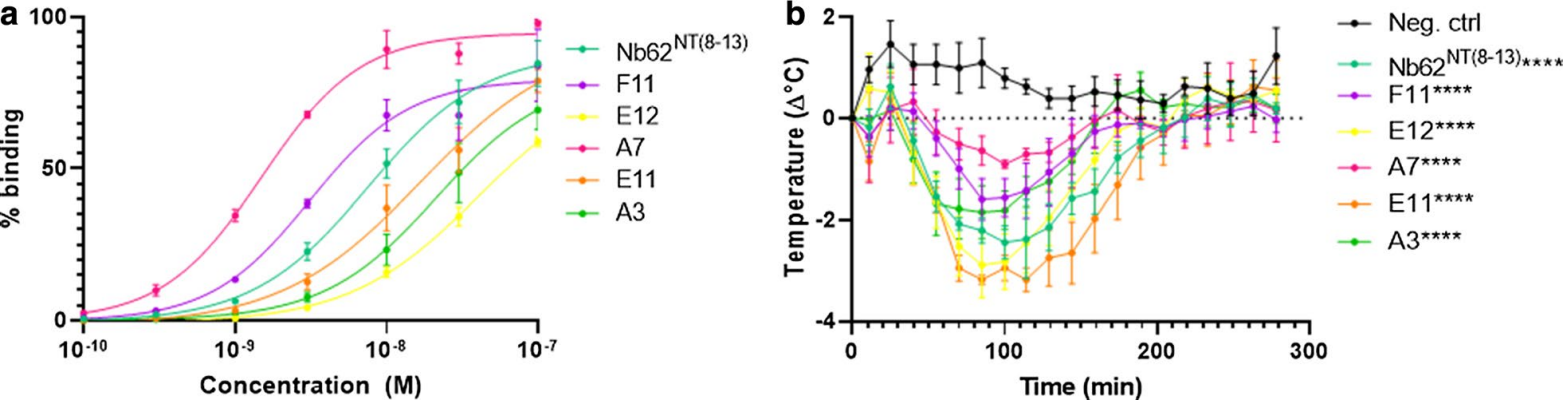
Brain-penetrating efficiency of Nb62 mutants. **a** Binding curves of anti-mTfR nanobodies to immobilized mTfR by ELISA (n = 4). Data were fitted by nonlinear regression using the GraphPad Prism 8 software (San Diego, CA, USA). **b** TLR4^−/−^ mice body temperature measurements after IV injections of Nb62 and mutants fused to NT (8–13) at a dose of 250 nmol/kg (****p < 0.0001). Bar graphs represent mean ± SEM (n = 3 per group). Statistical test: two-way ANOVA with Dunnett multiple comparisons test compared to negative control nanobody

A panel of Nb62 variants was generated by site directed mutagenesis to select the most efficient brain-penetrating nanobody. Their binding curves were generated by ELISA (Fig. 4a). Next, the nanobodies, fused to the catalytic amino acids of NT (8–13), were administered via IV injections at a dose of 250 nmol/kg and the body temperature of the mice was followed. As can be seen from Fig. 4b, the mutants give different body temperature profiles, indicating differences in brain uptake.

### Delivery route alters brain exposure

**Fig. 5:**
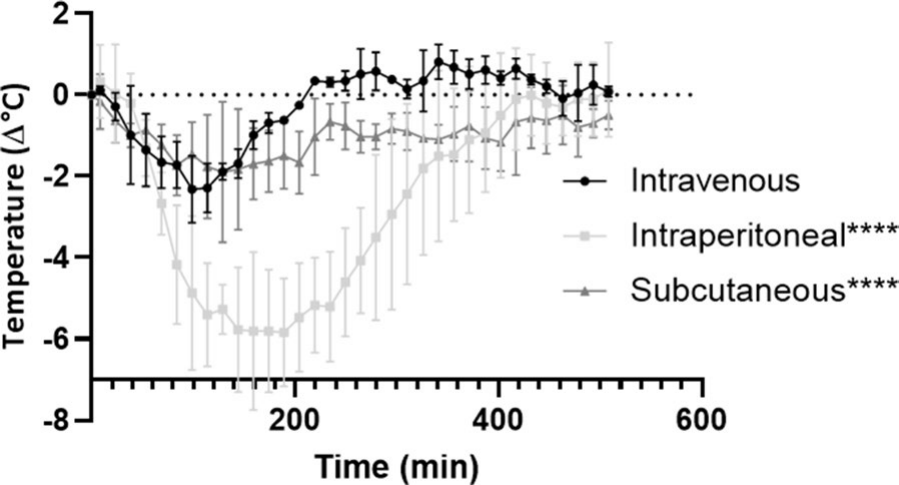
Effect of delivery route on brain exposure to Nb62. TLR4^−/−^ mice body temperature measurements after IV, IP or SC injections of Nb62 at a dose of 250 nmol/kg (****p < 0.001). Bar graphs represent mean ± SEM (n = 3 per group). Statistical test: two-way ANOVA with Dunnett multiple comparisons test compared to intravenous injection

We showed that the NT effect on body temperature is positively correlated to the dose present in the CNS. We next investigated which peripheral delivery route would provide the highest brain exposure. As can be seen from Fig. 5, intraperitoneal (IP) doubled the NT effect in terms of temperature drop and duration of the effect compared to IV injection, while the drop in body temperature starts later compared to IV or SC administration. This shows that delivery of Nb62 intraperitoneally is slower, but leads to more uptake in the brain.

## Discussion

Here, we describe the discovery and characterization of the first anti-mTfR nanobodies that enter the CNS from the periphery via receptor-mediated transcytosis demonstrated by a novel, robust in vivo validation method. This method allows animal re-use and relies on target-engagement by targeting the NTSR1 expressed on hypothalamic neurons. Nanobodies coupled to NT that are able to penetrate the brain will activate the NTSR1, which causes a drop in body temperature. This hypothermic effect has been described previously as a secondary effect following intravenous administration of ANG2002, which consists of NT fused to a brain-penetrant peptide Angiopep-2, targeting the LDL receptor–related protein-1 [50]. Next to the central hypothermic effect, Demeule et al*.* also observed in rats a blood pressure reduction following peripheral NT administration. Potentially, this peripheral effect of NT might also be present in mice and could potentially influence the body temperature of the animals. However, no drop in body temperature was observed for our non-BBB crossing controls which were fused to NT. Therefore, the observed temperature drop is centrally mediated and makes NT an ideal tool to validate BBB-crossing of agents like shown by this study. Even though the hypothermic effect of NT has been described before, this is the first time this robust and unambiguous model is used to rank multiple nanobodies in terms of their brain-penetration efficiency. These first anti-mTfR nanobodies are useful tools to study drugs that are targeted to brain targets and are unable to reach the CNS on their own, in a non-invasive way. Moreover, a brain-penetrating nanobody fused to NT itself could be a potential drug candidate in various diseases where body temperature lowering could be beneficial [52–55]. An example is induced hypothermia in acute ischaemic stroke, where a quick, but short duration of hypothermia is beneficial on infarct size [56].

In order to select brain-penetrating nanobodies by receptor-mediated and not adsorptive-mediated transcytosis, an acidic peptide was incorporated between the nanobody and NT sequence, which resulted in only neutral or acidic pI values (Fig. 2a). Next, their brain-penetrating capacities were determined. Out of the 7 nanobodies tested in vivo, only one was able to reach the brain parenchyma. There are several explanations possible why most of the anti-mTfR nanobodies do not cross in vivo*.* For instance their epitopes might be shielded in vivo by transferrin which has a micromolar plasma concentration and a nanomolar affinity for its receptor [57]. It is known that binding to the apical domain of the TfR can lead to brain uptake of monoclonal antibodies [31]. Here we show this also to be the case for the anti-mTfR nanobodies, which aligns with other agents reaching the brain via the TfR [4, 31, 58].

Next, the hypothermic effect was assessed for dose dependent effects. Different amounts of NT were injected in the lateral ventricles of mice. Higher doses resulted in deeper and longer drops in body temperature (Fig. 3a). Subsequently, Nb62 was injected intravenously with increasing dose. Again, the amount of injected nanobody corresponded with the hypothermic effect. The negative control did not elicit a drop in body temperature at the highest dose, showing there is no passive transport of acidic nanobodies at a dose of 500 nmol/kg. These experiments provide evidence that higher concentrations of brain-penetrating nanobody in the blood leads to more nanobody entering the brain and consequently a bigger NT effect.

Subsequently, we performed a limited structure–function analysis of Nb62 using our new in vivo screening method to identify more efficient brain-penetrating nanobodies. Here, mutants of Nb62 were generated with different binding affinities, since it is known that affinity for the mTfR affects brain uptake [27, 59]. By using site-directed mutagenesis, single site saturation libraries were generated where each amino acid in the CDR3 region of Nb62, which is most probably involved in antigen binding [60], was substituted to all other amino acids. The nanobodies, fused to the smallest active fragment of neurotensin (NT8-13), were expressed and purified, followed by the generation of binding curves by ELISA (Fig. 4a). The single amino acid substitutions resulted in a batch of nanobodies with different binding profiles. Next, the nanobodies were intravenously injected. As can be seen from Fig. 4b, different body temperature drop profiles were observed, indicating differences in brain uptake. Generally, we see that the nanobodies with the strongest binding have the lowest brain uptake. This is in accordance with literature regarding bispecific antibodies targeting the TfR, where it is shown that high affinity monoclonal antibodies are being located to the lysosomes for degradation [61]. In that paper, however, only two monoclonal antibodies were analyzed. All of the analyzed mutants in Fig. 4 induced a significant decrease in body temperature compared to the negative control, indicating they all penetrated the brain to some extent.

Finally, we tested different administration routes for injection of Nb62. IV, IP and SC injections give different PK profiles [62, 63], as can be seen from the shapes of the temperature curves in Fig. 5. By comparing the three administration routes, CNS delivery is highest upon IP injection, while a more continuous delivery is reached after SC injection. This is in line with literature where they rely on TfR targeting to deliver an anti-tumor necrosis factor decoy receptor antibody to the brain [63]. These differences in CNS delivery profiles can be contributed to the short plasma half-life of 10–20 min of nanobodies due to fast renal clearance [64, 65]. An immediate high blood concentration following IV injection might saturate the TfRs present at the BBB, which will deliver the VHHs to the brain parenchyma. Upon recycling to the luminal side of the plasma membrane, most of the VHH has been cleared from the bloodstream, resulting in the IV profile observed in Fig. 5. IP injection might also saturate the TfRs, but due to the sustained release it is possible for the recycled TfRs to be saturated again. This would lead to a double dose reaching the brain compared to IV, which is indicated by the doubling of the temperature drop and duration of the effect (Fig. 5). SC delivery leads to the lowest plasma concentration [63] which would not saturate the TfRs. However, the sustained release leads to a prolonged brain uptake compared to IV delivery. This is indicated by the plateau of the SC injection profile (Fig. 5).

The short half-life of nanobodies can be interesting for applications such as imaging [22] and PET/CT assessments [66], and can be prolonged for therapeutic applications by fusion to proteins where the nanobody adopts the half-life of the fusion protein, such as the Fc portion of a monoclonal antibody [67, 68]. Ultimately, next to being a tool to assess the brain-penetrating potential of novel RMT targets, nanobody-NT fusions have the potential to improve the speed of cooling acute stroke patients compared to conventional methods. Here, the short plasma half-life would be beneficial, since an inverse relation between the duration of hypothermia and infarct size is observed [56].

## Conclusion

In conclusion, we have discovered a first set of nanobodies that are able to deliver a payload to the brain via receptor-mediated transcytosis using the transferrin receptor. Simultaneously a novel in vivo platform was set-up to confirm brain penetration of nanobodies in an unambiguous manner. This method is able to directly compare different nanobodies in terms of their brain-penetrating potential. Moreover, the same method was used to show that highest brain uptake was obtained following intraperitoneal delivery. The use of this method could potentially be expanded to other less validated or novel receptor-mediated transcytosis targets to assess their capacity of potential therapeutic delivery towards the brain.

## Data Availability

The datasets used and/or analyzed in the current study are available from the corresponding authors upon reasonable request.

## Abbreviations

Aβ: Amyloid-β
BACE1: Beta-secretase 1
BBB: Blood–brain barrier
CNS: Central nervous system
ELISA: Enzyme-linked immunosorbent assay
ICV: Intracerebroventricular
IP: Intraperitoneal
IV: Intravenous
NT: Neurotensin
pI: Isoelectric point
RMT: Receptor-mediated transcytosis
mTfR: Mouse transferrin receptor
TLR4: Toll-like receptor
